# The Institutional Worker

**Published:** 1913-04-26

**Authors:** 


THE
### INSTITUTIONAL WORKER
Being a Special Supplement to "The Hospital"

### Stamping Receipts for Charitable Contributions.

It is not customary to stamp receipts for charitable contributions of £2 and over, though, if the donor insists upon it, you may find that it is policy to do so. Whilst there is no actual exemption by law, the omission of the receipt stamp is allowed by the Inland Revenue authorities.—H. F.

### A Good Heating System.

Experience seems to indicate that one of the best methods of heating hospitals is the low-pressure hot-water system. A system such as this, having an accelerated circulation, an advantage possessed by the Reck Patent Circulator system, is considerably more efficient than a low-pressure system which is entirely dependent for movement on gravity. Though there are other excellent systems, this one has been in use on the Continent and in this country for many years, and has been proved to be eminently satisfactory. The cost of installation and upkeep is somewhat less than ordinary hot water, and it can be combined with almost any system of ventilation. — Engineer.

### Convalescent Home for Child.

We should suggest you should apply to St. Joseph's Convalescent Home, Branksome Wood Road, Bournemouth, for the case you mention of a child aged 12 who needs change of air after pneumonia. This Home receives children as well as adults, and their charge for children under 12 is 6s. per week. —H. F. S.

### The Prospects of a Small Nursing Home.

Kindly read our rules, and comply with them; we cannot give postal replies. We would strongly urge you not to carry out your proposal; the prospect of success is doubtful, and it might lead to further disappointment. It would be better, under the circumstances, for you to endeavour to get a permanent appointment as a district or village nurse; in this matter the Rural Midwives' Association could, no doubt, assist you. Apply to the Secretary of the Association, 47 Victoria Street, London, S.W.—A. M.

### Maternity Practice.

It is somewhat difficult to commence a practice of this description in any district unless you are sure of the support of the medical practitioners. Unless you can select a place where you are either well known or are confident of securing the support of the profession, we would advise you to abandon the idea in favour of a salaried post. In the latter case the Rural Midwives' Association, 47 Victoria Street, London, S.W., would gladly help you.—L. F. B.

### Ambulance Classes.

Kindly read the rules and comply with them; we cannot give postal replies. You could obtain the experience you require by joining an ambulance class. Full particulars of such a class in Dublin may be obtained upon application to the Local Secretary, Dr. W. C. Stevenson, 60 Lower Baggot Street, Dublin.—L. H.

### How to Become a Male Nurse.

With the exception of a few men trained by one or two of the large London hospitals for their own service, the only civil hospital which trains male nurses is the National Hospital for the Paralysed and Epileptic, Queen Square, Bloomsbury, London, W.C. Candidates between the ages of twenty-one and thirty-five years, of good character, sufficiently well educated, and otherwise suitable, are accepted as probationers for a period of two years, subject to the regulations of the hospital. Application should be made to the Secretary at the hospital. This matter is dealt with very fully in the handbook published by The Scientific Press, Ltd., "How to Become a Nurse," by Sir Henry Burdett.—S. R.

### Needlework Guild.

The duties of the President of a Needlework Guild cannot be dogmatically defined. It is important that a lady should be appointed as president whose personality and local influence will attract support to the movement. Whilst a lady suitable for this position might not have the time to carry out many fixed duties, she could do much useful propaganda work whilst discharging her social obligations. It is desirable that she should preside at all important meetings, and that generally she should maintain the enthusiasm of the workers. The vice-president might be her deputy, but it would be probably better that a number of vice-presidents should be appointed—ladies willing to

### OUR BUREAU OF INFORMATION.

### Rules for Correspondents.

1. Every letter, on whatever subject, must be accompanied by the coupon to be cut from the back cover (inside page) of The Hospital, current issue, and must contain the name and address of the correspondent with pseudonym for publication if desired.
2. Replies by post cannot be given, save under exceptional circumstances at the Editor's discretion.
3. Letters from Approved Homes in reply to special needs published in the Bureau should state terms and full particulars, and be sent prepaid under cover to the Editor of the Bureau with name written across coupon for identification.
4. Proprietors of Homes which have not yet been entered on the List of Approved Homes, but have spare accommodation likely to suit special needs, are invited to write for an application form for registration. The fee for registration, which includes two announcements of the Home in the Bureau and other privileges, is 10s.
5. All communications to be addressed to the Editor of The Hospital, 28 Southampton Street, Strand, London, W.C., and marked "Bureau of Information."

### Invitation to Readers.

**The Editor is prepared to make known without charge the needs of any who may be sick or in difficulties, and to guide them in making choice of Homes for treatment or convalescence. Reduced terms can often be arranged with Homes on the Approved List for those unable to pay full fees. Queries are specially invited from those who are engaged in any kind of philanthropical work. A new edition of the leaflet describing the purposes of the Bureau of Information is now ready and will be sent free of charge on application.**

### THE COST OF LAUNDRY AT A LARGE PROVINCIAL HOSPITAL.

"Institutional" sends us the following complete and comprehensive answer to the April question in our Facts and Figures Department. The information it contains, being based on the actual working of a large hospital laundry, cannot fail to be of considerable value to other institutional workers. We commend this answer to their attention, both for the value of the information and the manner in which it is presented, and hope it will encourage others to send us replies to this question.

### Figures for Four Weeks of Winter Months.

The hospital to which these figures relate contains 430 beds, with an average number in daily occupation of 417. The classes of patients received are general, medical, and surgical, and there are about sixty beds for special departments. During the four weeks taken the average number of 417 was made up as follows: General surgical, 191; general medical, 166; skin, 15; gynæcological, 15; ophthalmic and throat and ear, 30. The number of garments washed was 65,722; the cost of laundry was £190 15s. 0¾d., giving a cost per piece washed of .69d.

### How the Cost has been Arrived at.

The above cost of the laundry department is arrived at on the following basis: The wages of laundry hands is the actual amount of wages pay a certain subscription, and to enlist the services of a number of associates and members. The secretary should discharge the usual executive duties, such as convening meetings, correspondence, and so forth. A very successful Linen League was formed at the Walsall Hospital a few years ago, and, although there are many such leagues, this is a good example on the smaller scale you mention, and the Matron would gladly furnish you with particulars upon application.—V. Y.

**Sick-room Help for the East-End Poor.**

Attendance upon poor women in the East End of London during sickness and confinement is provided by the Sick Room Help Society. This Society, which is partly supported by the weekly payments of those dependent upon it, secures for its members who have paid up 10s. a trained visiting nurse during sickness or confinement, as well as the attendance of a sick-room help, who remains in attendance all day from ten to fourteen days, caring for the family and carrying out the necessary domestic duties during the illness of the mother. Application for particulars of membership should be made to the Secretary, 24 Underwood Street, Vallance Road, Whitechapel.—District Visitor.

**Pensions for Incapacitated London Workers.**

The City of London General Pension Society grants pensions, amongst others, of 31s. a month to men who have been employed for twenty years or over in, or within twelve miles of, the City. Unless totally incapacitated, only those who are not less than sixty years of age are eligible. Candidates must be recommended by a Governor or subscriber, and an election of pensioners takes place once a year, or at such times as the funds and prospects of the Society permit. Full particulars may be obtained upon application to the Secretary, 12 Finsbury Circus, London, E.C. — Enquirer.

**Catholic Prisoner's Aid Society.**

Make your want known to the Catholic Prisoner's Aid Society, 57 Horseferry Road, Westminster, S.W. This Society assists Catholic prisoners by seeking employment for them, and by supplying them in the meantime with clothing, maintenance, and other necessaries. It also arranges for the visitation and relief of the Catholic members of distressed families of prisoners.—A. M. D.

paid to laundress, her assistants, laundry maids, and the laundry man. To this is added the board of the laundress and her two assistants, together with the estimated cost of food supplied to the laundry maids and the laundry man, who do not reside. The cost of uniforms supplied to laundry workers is also charged against the department. There is also a charge made against the laundry for the value of time spent on the laundry by the engineer, joiners, stokers, etc. Materials and sundries supplied by the various departments, such as soaps, soap powders, starch, blue, netting bags, felt, calender felt and sheeting, various items of hardware, crockery, and stationery, laundry baskets, matches, etc., are all charged against laundry account. As regards fuel, light, and power, an estimation is made of the amount of boiler coal that should be charged. The gas consumed is on a special meter, as also is the electric light, so that the exact cost is debited. Water also is on a special meter, and the quantity consumed is charged at the contract rate. Repairs to laundry buildings and laundry machinery and plant: a strict account is kept of all repairs done and all materials bought for purposes of laundry repairs. Depreciation is charged on the diminishing value of the laundry buildings at the rate of 1½ per cent. per annum, and on the diminishing value of the laundry machinery and plant at the rate of 7½ per cent. per annum. Interest is also charged against the department for the outlay of capital on both buildings and machinery. A proportion of the hospital rates is also debited against the department. Any special cleaning of the laundry, painting of walls, etc., that may be done: an estimation of the cost of material used and time spent is made, and is charged to the laundry account.

**The Amount of Washing Materials Used.**

The soap and soap powder used in the laundry during the four weeks taken was 8 cwt. laundry curd, costing £10; 3 cwt. soap powder, £2 5s.; 3½ cwt. crystal carbonate, 16s. 4d.; making the total cost of these items £13 1s. 4d. The quantity of starch and blue consumed was 40 lb. compound starch, costing 10s.; 336 lb. H.W. starch, £2 15s. 6d.; making the total cost of these items £3 5s. 6d., or a total cost for soap, soap powder, starch, and blue of £16 6s. 10d.

### Institution Facts and Figures.

### April Question.

Stating the number of beds in your institution and the class of patients received, give the following particulars:—

The number of garments washed during any four weeks in the winter months, with the price per unit, and the manner in which the cost is arrived at. In addition to wages, fuel, light and power, interest on capital should be taken into account. State the quantity, description, and cost of soap used in laundry for the same month. If curd and oil soap be used, the amount of alkali should also be stated. State also the quantity of starch and blue.

The best answers will be published, and for each contribution considered by the Editor to be of sufficient interest for publication payment of Five Shillings will be made.

All letters to be addressed, with coupon enclosed, to The Editor, The Bureau of Information, 28-29 Southampton Street, Strand, London, W.C., and to be received by the last day of the current month.

**Retreats for Men Suffering from the Drug Habit.**

Kindly read our rules and comply with them; we cannot give postal replies. We would suggest that you should apply to one of the following Homes, which are licensed under the Inebriates Acts; they also undertake the treatment of men suffering from the drug habit. Abbotswood House, Cinderford S.O., Glos. (apply Secretary, C.E.T.S., 4 The Sanctuary, Westminster), weekly fee from 10s. to £2 2s.; Dr. E. Norton, Capel Lodge, near Folkestone, three guineas; Dr. F. S. D. Hogg, Dalrymple House, Rickmansworth, from two to four guineas; Dr. G. Melville Smith, Buntingford House, Buntingford, Herts, from two to three guineas; Dr. J. W. Astley Cooper, Ghyllwoods, near Cockermouth, from three guineas. As a general rule the patient has to sign a form of consent that he will enter the home for treatment.—H. J.

**Home Wanted.**

A trained nurse, who has had heart trouble and has broken down in health, is in need of a home in a suitable institution where a rest cure, combined with medical supervision, could go on for about a year, and where she would be received free or at a very low figure. Her case is diagnosed as mitral stenosis, and the doctors consider that, after a long rest, she may be able to take up easy work connected with nursing. A home either at the seaside or the country would be suitable, and North Wales or the north-west coast of England would be preferable on account of distance. Will any of our Approved Homes or any charitable institution which can receive such a case reply to J. T. G.? (159A). Failing a home being found in the locality desired, we recommend that application should be made to the hon. secretary of the King Edward Memorial Home, Clapham Common; or to the Lady Superintendent, Filstone House, 26 Bloomsbury Place, Brighton. This latter is a home of rest, and it is possible the case in question may not be medically suitable.

### The Editor's Letter-Box.

**Communications have been received and attended to from:—**

Miss Appleton, Miss U. V. Townsend, Miss Spencer, and Miss F. R. Ward.

National Blind Relief Society (Rev. J. Pullein-Thompson).—We gratefully acknowledge receipt of donation of £1 for "Blind Ward Maid."

### Editor's Notices.

**Contributions:**

Contributions should be written, or preferably typed, on one side of the paper only, and all articles sent in are accepted upon the distinct understanding that they are forwarded to The Hospital only.

The Editor cannot undertake to return MSS. not used, but when a stamped directed envelope is enclosed the MSS. may be returned if a special request is made.

Accepted articles and paragraphs of news will be paid for after publication at the scale rate.

**Address:**

To prevent delay all contributions and letters on editorial business must be addressed exclusively to the Editor, "The Hospital," 29 Southampton Street, Strand, London, W.C. It is important that this regulation shall be strictly observed.

**Correspondence:**

Correspondence on all subjects is invited. The name and address of correspondents must be given as a guarantee of good faith, but not necessarily for publication.

**Special Articles:**

Special articles are invited, and questions, inquiries, and paragraphs upon all matters relating to the work, administration, and management of general, special, mental, fever, cottage and convalescent hospitals, sanatoria, homes, institutions, societies and organisations for the treatment or care of the sick, injured and dependants of all classes. Every matter affecting the interests and welfare of the staff of all grades working in these institutions will receive special consideration.

**Administrative Medicine, Research and Items of News:**

Special terms will be paid for approved articles on subjects in this wide field by experts who have made some section of it their special study and interest. We wish to give prominence to every movement tending to advance accuracy of diagnosis, efficiency in treatment, and the development of modern methods to eradicate disease and promote the welfare of the suffering.

**A Bureau of Information:**

We invite inquiries and applications for information and help in providing for that numerous class of sufferers who require special care or house-room which they cannot obtain in their own homes or secure for themselves. We cannot, however, prescribe, or recommend practitioners.

**Books for Review:**

Publishers are particularly requested to send advance proofs of any new books of importance whenever possible, as the Editor has made arrangements to publish immediate reviews on a new plan.

**Photographs, Plans, Blocks, and Illustrations:**

It is requested that wherever possible MSS. may be accompanied by illustrations in any of the above forms. The name of the sender and of the article to which it belongs should be written on the back of each photograph, drawing, or block for purposes of identification.

**Local Papers:**

Newspapers containing reports or news paragraphs should be marked and addressed to the Sub-Editor.

**The Coupon System:**

A coupon will be found at the bottom of the third inside page of the cover each week. This coupon must be attached to every question or inquiry to which an answer is desired in The Hospital.

### A Help to the Worker.

As a means of announcing vacancies in every department of Institutional work, the success of *The Institutional Worker* is evidenced by the ever-increasing number of advertisements that appear in this section of The Hospital. No more adequate proof of the value of *The Institutional Worker* can be desired than the fact that Authorities of the more important Institutions throughout the United Kingdom employ its columns again and again whenever they have a vacancy to fill upon their staffs. Moreover, it provides exceptional facilities for every class of announcement affecting Institutional Life and Work, and as a medium for securing appointments in any section of the Institutional, Health, and Sanitary Services it is a distinct help to the worker, since no other journal possesses such a comprehensive circulation in these directions as The Hospital. If you are seeking a post an announcement of twenty-one words, costing the small sum of 1s., will save you many hours of anxious thought, and certainly bring your requirements immediately to the notice of those most likely to need your services.

The form below may be used for Appointments Wanted advertisements, and when filled up should be posted to the

*Manager*, The Hospital,
28 and 29 Southampton Street,
Strand, W.C.

Appointments Wanted, 21 words ... 1s. 0d.

| | | |
|---|---|---|
| | | |
| | | |
| | | |
| | | |
| | | |
| | | |
| | | |

### Manager's Notices.

**Letters relating to the publication, sale, and advertising departments of "The Hospital" must be addressed to The Manager, "The Hospital," The Hospital Building, 28 and 29 Southampton Street, London, W.C.**

**Advertisements:**

To ensure insertion, all advertisements for The Hospital must reach the Manager not later than Wednesday morning in each week. For scale of charges see pages iii and 4.

**Subscriptions** may begin at any time, and are payable in advance. Cheques and Post-Office Orders should be crossed London County and Westminster Bank, Covent Garden Branch, and made payable to the Manager of The Hospital.

Rates of Subscription (including Postage).

| *United Kingdom.* | | *Foreign and Colonial.* | |
|---|---|---|---|
| Three Months ... ... | 2s. 0d. | Three Months ... ... | 2s. 6d. |
| Six Months ... ... | 4s. 0d | Six Months ... ... | 5s. 0d. |
| Twelve Months ... ... | 6s. 6d | Twelve Months ... ... | 8s. 8d. |

**Remittances:**

**It is especially requested that remittances be made by Cheque or Postal Order, and in halfpenny stamps only when the amount is under sixpence.**

